# The Effector Function of Allergens

**DOI:** 10.3389/falgy.2022.818732

**Published:** 2022-02-07

**Authors:** Stéphane Hazebrouck, Nicole Canon, Stephen C. Dreskin

**Affiliations:** ^1^Université Paris Saclay, CEA, INRAE, Département Médicaments et Technologies pour la Santé (DMTS), SPI, Gif-sur-Yvette, France; ^2^Division of Allergy and Clinical Immunology, Department of Medicine, University of Colorado Denver, Aurora, CO, United States

**Keywords:** allergens, effector function, fcepsilonR1, IgE, mast cells, cross-linking, epitope, degranulation

## Abstract

Allergens are antigens that generate an IgE response (sensitization) in susceptible individuals. The allergenicity of an allergen can be thought of in terms of its ability to sensitize as well as its ability to cross-link IgE/IgE receptor complexes on mast cells and basophils leading to release of preformed and newly formed mediators (effector activity). The identity of the allergens responsible for sensitization may be different from those that elicit an allergic response. Effector activity is determined by (1) the amount of specific IgE (sIgE) and in some circumstances the ratio of sIgE to total IgE, (2) the number of high affinity receptors for IgE (FcεR1) on the cell surface, (3) the affinity of binding of sIgE for its epitope and, in a polyclonal response, the collective avidity, (4) the number and spatial relationships of IgE binding epitopes on the allergen and (5) the presence of IgG that can bind to allergen and either block binding of sIgE and/or activate low affinity IgG receptors that activate intracellular inhibitory pathways. This review will discuss these important immunologic and physical properties that contribute to the effector activity of allergens.

## Introduction

The word allergen refers to a specific molecule, usually a protein that generates an IgE response as opposed to a more general term, antigen that is a molecule that induces any immune response (1). Allergenicity can refer to the ability of an antigen to elicit the production of specific IgE (sIgE), bind to sIgE antibodies, induce cross-linking of sIgE bound to the high affinity receptor, FcεR1, for IgE (IgE/FcεR1complexes) and trigger cell degranulation that may ultimately lead to an allergic reaction in a sensitized subject (2, 3). Arguably, all of these aspects must be present for an allergen to be clinically relevant although there are exceptions due to cross-reactivity when IgE sensitization is primarily induced by a different allergen (2, 3). The concept of “allergenicity” in general or in a specific context has been reviewed previously, most often focusing on the ability of allergens to elicit an IgE response but also acknowledging the importance of clinical reactions (4–16). This review is focused solely on the effector function of allergens and the ability of an allergen to elicit an allergic reaction by cross-linking of IgE/FcεR1complexes to activate mast cells and basophils. The examples given are frequently regarding peanut allergens as these have been extensively studied. However, these concepts likely apply to most, if not all, allergens.

Before discussing the effector function of allergens, it is important to note that the allergens within an allergenic source (e.g., a food) that are the most potent for sensitization may be different from the allergens that are responsible for elicitation of the allergic response. As an example, sensitization to peanuts may be mediated primarily by Ara h 1, which is a vicilin, also known as a 7S globulin (17, 18). In another mouse model, IgE sensitization to Ara h 1 was induced after oral sensitization to raw or roasted peanut (19). In contrast, production of IgE to Ara h 6 was not induced in mice sensitized to raw peanut and purified native Ara h 6 also displayed limited intrinsic sensitization capacity (19). The formation of large complexes during heat-treatment, notably between Ara h 1 and Ara h 6, were probably required for Ara h 1 to act as carrier for Ara h 6 uptake, activation of dendritic cells, and induction of specific Th2 immune response to Ara h 6. On the other hand, the 2S albumins of peanuts, Ara h 2 and Ara h 6, have been shown to be much more potent than Ara h 1 or Ara h 3, an 11S-globulin, for eliciting IgE-mediated mast cell activation in peanut allergy (16, 17, 20–22). The mechanism whereby one allergenic protein may sensitize and thus generate an IgE response that allows a different but homologous allergen to cause an effector response, likely involves cross-reactive linear and/or conformational epitopes (23, 24). However, for proteins displaying low overall identity such as between Ara h 1 and Ara h 2 or between peanut and tree-nut proteins, the mechanism is less well-understood and may involve discrete sequences similar in physicochemical properties (16, 25–27).

Allergenic proteins are limited to a small number of protein families (28). In some cases, the effector function is predominantly mediated by even a smaller number of allergens that can be specifically depleted by specific high affinity antibodies. This has been tested for peanut allergens, where, following immunodepletion, Ara h 2 and Ara h 6 were found to account for most of the allergenic activity of a peanut whole extract (22, 29). Similarly, immunodepletion of Fel d 1 from an extract of cat allergens demonstrated that Fel d 1 is the most potent cat allergen (30). On the other hand, other allergenic extracts appear to have multiple allergens that contribute to effector function with combinations that vary in clinical relevancy for different individuals (31).

Thus, the ability of an allergen to cross-link IgE/FcεR1complexes on mast cells and basophils is determined by (1) the amount of specific IgE (sIgE) and the ratio of sIgE to total IgE (tIgE), (2) the number of FcεR1 molecules on the cell surface, (3) the affinity of binding of sIgE for its epitope and, in a polyclonal response, the collective avidity, (4) the number and spatial relationships of IgE binding epitopes on the allergen and (5) the presence of IgG that can bind to allergen and either block binding of sIgE and/or activate low affinity IgG receptors (FcγR2 in humans and FcγR2b in mice) that activate intracellular inhibitory pathways (32–36).

## Total and Specific IgE

The term specific IgE has evolved over the years. This was initially referring to IgE detected when a saline extract of an allergenic source such as individual animal danders, specific pollens, mold spores or food was used as the capture in an absorbent assay. More recently, specific IgE refers to IgE that binds an individual allergenic protein such as Fel d 1 from cat or Ara h 2 from peanut as discussed in this review. sIgE can be measured in research labs using ELISA assays and commercially in the ImmunoCap® (ThermoFisher) or other similar format. Microarray technology (*e.g*. ISAC® by ThermoFisher) has allowed simultaneous measurement of IgE binding to a variety of purified or recombinant allergens (37).

As the Cε3 region of the Fc portion of IgE binds to the alpha chain of FcεR1, there is equal binding of all IgE molecules to FcεR1, independent of specificity which resides in the Fab region. However, the density of any given specific IgE on the cell surface is inversely proportional to the amount of IgE of other specificities. Therefore, the effector activity of an allergen is influenced not only by the number of IgE/FcεR1 complexes but also by the ratio of sIgE to tIgE. This becomes important when considering sIgE in a sample with a substantial portion of the IgE directed at other allergens or without known specificity. Consequently, if the ratio of sIgE to tIgE is high, the probability of the same allergen to cross-link with sIgE and meet the threshold of complexes required to generate a measurable effector response is easier to reach than if the ratio of sIgE to tIgE is low (see below). Blanc et al. showed that sera exhibiting a lower ratio sIgE to tIgE required higher concentrations of allergens to trigger RBL SX-38 degranulation, which was not efficiently induced when the ratio was <2% (21). However, using human cultured mast cells, the lowest fraction of sIgE able to activate cells was around 0.3% (38). Hemmings et al. reported that, for peanut allergens, the ratio of sIgE to tIgE, along with the diversity of the sIgE repertoire to peanut allergens, were the major determinants of basophil and mast cell activation (39). In that study, compared to the level of sIgE alone, the ratio sIgE to Ara h 2 and Ara h 6 to tIgE improved the discrimination between patients who were clinically allergic peanut as opposed to those who were only sensitized (39). In addition to the amount of sIgE, the affinity of that IgE for binding to allergens is important (34, 38). This is further discussed below.

## Effector Cells and FcεR1

The effector function of an allergen requires that sIgE be bound to FcεR1 on mast cells and basophils. While the half-life of circulating IgE is ~1 day (40), IgE bound to FcεR1, due to the slow off rate of this interaction (K_D_ of 10^−9^ M), remains on the cell surface for several weeks (41). The function of FcεR1 has been extensively studied on rat basophilic leukemia (RBL) cells using the 2H3 clone (34) and on human basophils (42, 43). Using a monoclonal IgE, crosslinking as few as 100 FcεR1-IgE complexes leads to measurable cell activation and 50% cell activation was seen with crosslinking of 300-2000 FcεR1-IgE complexes (42, 44). Whereas, the number of FcεR1 on the RBL-2H3 cells is stable, the expression of FcεR1 on human basophils is upregulated by the concentration of circulating IgE so that naturally occurring basophils can have from 5 X 10^4^ to 5 X 10^6^ receptors per cell (42). Of note, in addition to their capacity to up-regulate FcεR1 levels on the cell surface, IgE antibodies also enhance mast cell survival and expansion (45). The density of FcεR1 on the cell surface is thus important in assessing the allergen concentration capable to trigger mast cell degranulation.

An interesting caveat that has been recognized for some time is that some patients may produce IgE that has an increased propensity to be cross-linked but the molecular basis of this finding was not understood (46). Recently, characterization by mass spectrometry of serum IgE glycosylation revealed that tIgE from peanut-allergic patients have increased terminal sialylation compared to tIgE from non-atopic controls. Of note, IgE sialylation did not impact the binding of IgE to FcϵRI but was found to modulate the signaling downstream of the receptor (47). The general impact of this observation has not been established.

## Not All Allergens That Bind IgE Have Similar Effector Activity

Levels of sIgE can be evaluated through different approaches and assays such as immunoblots, ELISA and competitive inhibition of IgE-binding. However, IgE-binding does not necessarily correlate with relevant clinical activity. To date, 17 peanut proteins have been reported as allergenic by the WHO/IUIS Allergen Nomenclature Sub-Committee. Among them, four peanut allergens, Ara h 1, Ara h 2, Ara h 3 and Ara h 6 bind large amounts of IgE on immunoblots which is is true for the majority of patients (48, 49). However, when studied in functional assays, it was found that Ara h 2 and Ara h 6 account for 70–90% of the allergic effector activity of peanuts (50). In addition, desensitization with only a combination of Ara h 2 and Ara h 6 protected peanut allergic mice from anaphylaxis elicited by whole peanut extract (29). Moreover, although co-sensitization to both Ara h 2 and Ara h 6 generally occurs in patients, IgE cross-reactivity can lead to a high level of IgE-sensitization to both allergens, as determined by ELISA, whereas for some patients, only one of the two 2S-albumins can effectively trigger mast cell degranulation *in vitro* (51).

## The Contribution of Physical Stability and Structure to Effector Function

Inhalant allergens have immediate interactions with the respiratory mucosa and do not have to be particularly stable (12). For example, the PR-10 allergens are generally sensitive to heat and to digestion. Consequently, they often can elicit allergic reactions at mucosal surfaces of the respiratory tract but not of the digestive tract (52). On the other hand, food allergens must be stable enough to withstand the environment of the digestive tract. Moreover, to enter the systemic circulation, soluble allergens are more efficiently transferred across the intestinal epithelial barrier, through transcytosis, than particulate allergens, which are processed through Peyer's patches (53). Therefore, soluble allergens are more potent in triggering anaphylactic reaction while particulate allergens, like aggregates resulting from heat-treatment, food processing or gastric acidity, could be more potent in inducing IgE sensitization (53). For these reasons, physical stability and structural elements such as size, oligomerization, aggregation or formation of micelles are important attributes of food allergens that, when studied, can greatly affect their effector function (6).

For example, Ara h 2 and Ara h 6 are much more resistant to gastric acid and digestive enzymes than are Ara h 1 and Ara h 3. This resistance likely enhances their effector activity (54–60). Furthermore, digestion products of peanut 2S albumins yield a large and a small subunit (5 and 9 kDa) that remain associated by disulfide bridges so that the resulting heterodimer is as immunoreactive as the intact protein. Hence, immunoreactive Ara h 2 and Ara h 6 can be still detected and quantified in the bloodstream and in breast milk of non-peanut allergic human volunteers after peanut consumption or in the blood of peanut allergic patients after oral food challenge (61). These proteins have been shown to bind IgE and to be active in functional assays with RBL SX-38 cells (60, 62–66). On the other hand, the primary allergen in wheat induced anaphylaxis, ω-gliadin, although stable to digestion, is a larger 30–50 kDa protein (67, 68) that requires cofactors that either augment gut permeability or cell responsiveness to cause anaphylaxis (69–71).

## IgE-Epitope Interactions

In addition to the number of allergen-specific IgE molecules, the affinity of binding is important (34). The complex relationship between the IgE repertoire and IgE affinity has been recently explored (38, 72, 73) underscoring the importance of tIgE, sIgE, IgE binding affinity and the diversity of the epitopes that bind IgE (72–74). The extent of effector cell activation is also linked to an allergen's oligomeric state, and the valency, spacing/proximity and flexibility of the IgE-binding epitopes (75, 76).

Identification of the IgE-binding epitopes has typically focused on linear sequences as they can be exhaustively and easily characterized by using overlapping peptide libraries and high-throughput technologies (77, 78). Recent diagnostic developments have successfully used peptide microarrays and bead-based epitope assays for profiling epitope-specific IgE repertoire in the context of peanut allergy (79–82). However, conformational epitopes (formed by 3D-folding of the primary amino acid sequences of a protein) are now thought to be critical for high affinity IgE binding to a number of important allergens, including Ara h 1 (83, 84), Ara h 2 (85–87), Ara h 6 (85–87) and others (88–92). The importance of conformational epitopes has been clearly shown by disrupting the disulfide bonds that play an important role in maintaining the structure of the 2S albumins. Chemical reduction and alkylation of these disulfide bonds lead to a loss of secondary, tertiary and quaternary structures with concomitant changes in biochemical as well as immunological characteristics (77, 93, 94).

The importance of conformational structure for Ara h 2 and Ara h 6 was best demonstrated with several reports showing the loss of IgE binding and mast cell activation following reduction and alkylation (77, 94–97). For Ara h 6, IgE-binding was shown to be critically dependent upon conformational epitopes (87, 98). For Ara h 2, around three quarters of 48 peanut allergic patients recognized conformational epitopes to a similar or greater extent than linear ones (99). That said, the contribution of linear vs. conformational epitopes in the overall IgE-binding capacity of Ara h 2 is highly variable among patients (98).

Indeed, the IgE-binding capacity of the entire allergen could be recapitulated by combining a synthetic peptide containing the immunodominant linear epitope plus a mutant of Ara h 2 in which this immunodominant linear epitope had been deleted. That peptide, which is found within an unstructured surface loop of Ara h 2, contains the repeated linear motifs DPYSP^OH^S, for which IgE-binding is highly dependent upon the presence of hydroxyproline, a post translational modification that occurs commonly in nature (98). On average, IgE-binding to this linear motif account for half of the total IgE-binding capacity of Ara h 2. The remaining IgE binding activity is to conformational epitopes (51, 98).

High-affinity IgE antibodies may appear as a prerequisite because sIgE is 10^5^ times less prevalent than IgG. These IgE antibodies must compete with lower affinity sIgG to bind allergen and initiate the allergic response. Thus, it has been shown that basophil degranulation is enhanced when the allergen is first anchored at the surface of the effector cell by high-affinity IgE bound to the FcεR1. Low-affinity IgE may then contribute almost as efficiently as high-affinity IgE to cell activation when cross-linking occurs with the second IgE (100). Another example is found with the IgE binding to the unstructured region of Ara h 2 that is discussed above. Here, IgE-binding to free peptides found in this region occurs without the large loss of affinity usually observed with free peptide compared to the full allergen (100). This is probably due to an increased avidity caused by the repetition of the motif DPYSP^OH^S two- and three-times in the isoforms Ara h 2.01 and Ara h 2.02, respectively. Accordingly, the free peptide containing three DPYSP^OH^S motifs displayed a higher capacity to inhibit IgE-binding to nAra h 2 and a higher potency in mast cell degranulation than the one with a lower valency (98). Of note, the two motifs in the shorter peptide are separated by only one residue. In this regard, the influence of spacing between IgE epitopes was also investigated by using rigidly spaced bi- and trivalent haptens or artificial multivalent allergens (33, 36). In the latter case, a non-allergenic myoglobulin, in which 4 repetitions of the same IgE-reactive peptide were grafted and separated by a linker of 6 Gly residues, triggered RBL degranulation more efficiently than derivatives carrying only 2 repetitions or more distant IgE epitopes (76). In these settings, multivalency of allergens, spatial clustering of IgE epitopes on a particular segment of the allergen and high-avidity interactions can overcome the need for high-affinity interactions to trigger cell degranulation, thus offering an explanation for clinical reactivity induced by unexpected low-affinity IgE cross-reactivity among different allergens (75, 101, 102).

## IgG Regulation of Effector Function

IgG to allergens occur naturally and can be further induced by specific immunotherapy. This has been best studied in the field of food allergy. IgG induced by oral immunotherapy for food allergy can bind to allergen and either block binding of IgE and/or activate low affinity IgG receptors (FcγR2 in humans and FcγR2b in mice) that activate intracellular inhibitory pathways (79, 103, 104). So, the effector function of an allergen is affected not only by the amount and affinity of sIgE, but also by the presence of specific IgG that may bind to the same epitope as the IgE (blocking IgG) and IgG that may bind to different parts of the allergen and activate inhibitory pathways (104).

## Conclusion

The determinants of the effector function of an allergen are complex and are affected by multiple components (Table 1). These include binding characteristics such as the amount and affinity of the IgE that binds to the allergen, and the presence of either non-specific IgE or IgE of other specificities that may effectively dilute out the allergen-specific IgE. The intrinsic properties of the allergen such as its stability and surface structure (number, spacing and spatial relationships of IgE binding epitopes) also play a vital role. Other contributing factors include the presence of IgG that may either compete with the IgE for specific epitopes or may bind to other epitopes and activate inhibitory receptors. Cellular features such as the numbers of high affinity receptors for IgE may render the same IgE-allergen interaction more clinically severe in one patient compared to another.

**Table 1:** Determinants of the effector function of allergens.

**Factors**			**Features**	
Total and specific IgE	Concentration of sIgE	Ratio of sIgE to tIgE	Clonality sIgE repertoire Cross-reactivity	Affinity/avidity of sIgE
Effector cells and Fc R1	Mast cells Basophils	Homogenous cell lines vs heterogenous fresh cells	FcεRI density on cell surface	IgE sialylation
Biochemical (and biological) properties of allergens	Stability Resistance to digestion	Size globular vs unstructured or disordered	Monomeric vs multimeric	(Sensitizing vs eliciting)
Epitopes	Diversity	Conformational vs. linear	Post-translational modifications (hydroxyproline, disulfide bridges)	Spacing Clustering Orientation
IgG	Concentration of sIgG1	Activation of inhibitory receptors	Blocking of IgE binding	Affinity/avidity for allergen

## Author Contributions

All authors listed have made a substantial, direct, and intellectual contribution to the work and approved it for publication.

## Funding

This work was supported AlimH department of INRAE (SH) and by RO1AI165866 (SCD) and R21AI135397 (SCD).

## Author Disclaimer

Contents are the authors' sole responsibility and do not necessarily represent official NIH views.

## Conflict of Interest

SCD has received grant support from the National Institutes of Health. The remaining authors declare that the research was conducted in the absence of any commercial or financial relationships that could be construed as a potential conflict of interest.

## Publisher's Note

All claims expressed in this article are solely those of the authors and do not necessarily represent those of their affiliated organizations, or those of the publisher, the editors and the reviewers. Any product that may be evaluated in this article, or claim that may be made by its manufacturer, is not guaranteed or endorsed by the publisher.
